# Endothelial Keratoplasty Following Glaucoma Filtration Surgery: A UK Tertiary Eye Care Referral Centre Experience

**DOI:** 10.3390/jcm13206097

**Published:** 2024-10-13

**Authors:** Francesco Aiello, Francesco Matarazzo, Maria Phylactou, Kirithika Muthusamy, Vincenzo Maurino

**Affiliations:** 1 Ophthalmology Unit, Department of Experimental Medicine, University of Rome “Tor Vergata”, 00133 Rome, Italy; 2 Department of Neurosciences, Reproductive Sciences and Dentistry, University of Naples “Federico II”, 80131 Naples, Italy; 3 Department of Physics “Ettore Pancini”, University of Naples “Federico II”, 80131 Naples, Italy; 4 Cornea Service, Moorfields Eye Hospital, NHS Foundation Trust, London EC1V2PD, UK; 5 Nicosia Vision Clinic, Private Clinic, Ilia Venezi 2A, Strovolos, Nicosia 2042, Cyprus

**Keywords:** DMEK, Descemet stripping endothelial keratoplasty, fuchs dystrophy, trabeculectomy, glaucoma drainage implant

## Abstract

**Purpose:** To compare the postoperative complications and clinical outcomes of Descemet membrane endothelial keratoplasty (DMEK) and Descemet stripping automated endothelial keratoplasty (DSAEK) in eyes with previous glaucoma filtering surgery. **Methods:** In this retrospective comparative case series, we analysed postoperative visual acuity and intraocular pressure, graft survival, rate of graft detachment and/or dislocation, number of rebubbling and/or graft repositioning procedures, and graft rejection or failure (primary and secondary). **Results:** Sixteen eyes with DMEK and 80 eyes with DSAEK with previous glaucoma surgery were studied. The results were recorded at 3 and 12 months postoperatively. No statistically significant differences in postoperative visual acuity were found between the two groups at any stage of the follow-up. Intraocular pressure was lower in the DMEK group at the follow-up stage of 3 (*p* = 0.0022) and 12 months (*p* = 0.0480). Visually significant graft detachment was recorded in 31.3% and 22.5% of DMEK and DSAEK cases, respectively (*p* = 0.4541). All DMEK detachments (n = 5) were managed with slit-lamp rebubbling. Out of 18 graft detachments in the DSAEK group, 2 grafts were observed due to small graft detachment, 6 large graft detachments underwent rebubbling performed in the operating theatre, and 10 eyes needed primary graft repositioning for graft dislocation. **Conclusions:** DMEK is a feasible option to treat endothelial failure in complex eyes with previous glaucoma surgery. In the DMEK group, visual acuity outcomes and possibly postoperative intraocular pressure control were better compared with the DSAEK group.

## 1. Introduction

Endothelial keratoplasty (EK) has become the treatment of choice for endothelial dysfunction [1]. This type of surgery has revolutionised the management of corneal diseases affecting the endothelial layer, offering a more targeted and less invasive approach compared to older methods [1,2]. It has replaced penetrating keratoplasty due to better postoperative refractive outcomes and visual acuity, faster visual recovery, and reduced postoperative complications [2,3]. Descemet stripping automated endothelial keratoplasty (DSAEK) and Descemet membrane endothelial keratoplasty (DMEK) are the most widely used EK techniques worldwide. Both procedures involve the selective removal and replacement of the diseased endothelial layer, but they differ in terms of the technique and the thickness of the tissue graft used. DMEK has shown faster visual recovery, better visual outcomes, and similar endothelial cell loss compared with DSAEK [4,5]. Patients undergoing DMEK typically experience clearer vision sooner, with less chance of graft rejection, making it the preferred choice in many cases where it is technically feasible [4,5]. Despite this clear advantage, the global adoption of DMEK has been hampered as it represents a difficult procedure with a steep learning curve [6,7,8].

Performing DMEK surgery in eyes following glaucoma filtration procedures poses an additional challenge. These cases are more complex due to pre-existing alterations in the eye’s anatomy and function, which may complicate the surgical process and affect the outcomes. Several factors have been proposed as being able to complicate the procedure, such as a deep anterior chamber (AC) depth or the presence of an incomplete iris plane [2,9,10]. These anatomical variations can make it harder to position the graft correctly or to maintain the necessary pressure in the eye during surgery, increasing the chances of postoperative issues. Additionally, the presence of a glaucoma drainage device facilitates air dissipation through the filtering route, not allowing a satisfactory gas tamponade to be achieved. As a consequence, a proper graft attachment is less likely to be obtained due to a poor gas tamponade [2]. This significantly raises the risk of graft failure or the need for additional surgical interventions, making such cases particularly challenging to manage.

Hence, the risk of graft detachment and dislocation in such cases is higher and can lead to further intervention or to primary graft failure due to excessive intraoperative graft manipulation [11]. In our retrospective study, we aimed to compare the outcomes and the postoperative complication rates for DMEK and DSAEK at 12 months in eyes which had previously undergone glaucoma filtration surgery (trabeculectomy, tube shunt, or both). By evaluating these two techniques side by side in this specific patient group, we hoped to gain insights into the best practices for managing complex cases and optimising long-term visual outcomes.

## 2. Patients and Methods

We retrospectively analysed the clinical records of consecutive patients with a history of previous glaucoma filtration surgery (trabeculectomy, tube shunt, or both), undergoing endothelial keratoplasty at Moorfields Eye Hospital, London, UK. The patients’ notes were retrieved from an electronic healthcare record system (Open Eyes v1.18, www.openeyes.org.uk, accessed on 01 February 2018). The study period lasted from January 2012 to September 2017.

The study was approved as a clinical audit project by the Clinical Audit and Effectiveness Committee at Moorfields Eye Hospital (CA16/CED/13, approval date: February 2018). The tenets of the Declaration of Helsinki were followed with informed consent for surgery as part of the routine clinical care. The eyes were divided into 2 groups: eyes undergoing DMEK surgery (DMEK group), and eyes undergoing DSAEK surgery (DSAEK group). We recorded the preoperative demographics, ocular history, use of glaucoma medications, indication for EK, preoperative and postoperative best-corrected visual acuities (BCVA) and intraocular pressure, postoperative complications such as graft detachment and/or dislocation, graft rejection or failure (primary or secondary), pupillary block, additional postoperative procedures and, in particular, the number of re-bubblings or graft repositionings.

Visual acuity was measured using the Snellen meter then converted using the logarithm of the minimum angle of resolution (LogMAR) for statistical analysis.

Postoperative glaucoma raised intraocular pressure (IOP) was defined as IOP > 24 mmHg or a relative increase of >10 mmHg compared to the preoperative value.

We differentiated between graft detachment and graft dislocation. In the first case, the graft was only partially detached from the cornea. Graft dislocation was defined as the presence of a free-floating graft in the AC [12]. Graft detachment was further defined as small when involving less than 1/3 of the graft surface and large when involving 1/3 or more of the graft surface [12]. At our institution, DMEK slit-lamp graft rebubbling is usually performed in cases of a large graft detachment or a graft detachment involving the visual axis, causing visual deterioration [11,12].

Primary graft failure was defined as an oedematous cornea which failed to achieve deturgescence and required a re-graft within the first 8 weeks of surgery, in the absence of surgical complications [13]. Graft failure was defined as the irreversible loss of graft clarity [14].

Endothelial graft rejection was defined as evidence of AC cell activity, endothelial rejection line, or keratic precipitates with associated corneal oedema and the presence of inflammation [15].

All patients with a postoperative follow-up duration of less than 12 months were excluded from the graft survival analysis.

### 2.1. Donor Tissue

All the DMEK donor tissues were prepared in the operating room by the operating surgeon, a few minutes prior to surgery. The DSAEK tissues were prepared by the operating surgeons prior to surgery, until November 2015. Subsequently, all the DSAEK grafts were prepared on the day prior to surgery by the Moorfields Lion Eye Bank, using an artificial AC and a microkeratome.

### 2.2. Postoperative Care

The operated eyes were checked 2 h after surgery prior to the patient discharge to check for the presence of a pupillary block. The patients were then asked to posture face up to the ceiling for most of the time for the first 48–72 h post-surgery. Pupil dilation was maintained for 3 days when no iridectomy had been performed. Routine postoperative medication included Chloramphenicol 0.5% (Bausch & Lomb, Aubenas, France) eye drops 4 times daily for 2 weeks, and topical Dexamethasone 0.1% (Maxidex-SA Alcon-Couvreur NV, Puurs, Belgium) 1–2 hourly for 1 week, to be tapered in 12 months. Any glaucoma medication used by the patient before the surgery was continued after the EK procedures. All the patients had their first postoperative review within one week, with a variable follow-up regimen dictated by clinical progress afterwards. In the case of steroid-related glaucoma, the Dexamethasone 0.1% was discontinued and Fluorometholone 0.5% (Allergan Pharmaceuticals, Dublin, Ireland) or Loteprednolole 0.5% (Bausch & Lomb, Aubenas, France) was started. In these cases, the anti-glaucoma drops treatment was adjusted in accordance with glaucoma specialist advice.

### 2.3. Outcome Measures

The main outcomes were postoperative visual acuity, postoperative IOP, the number of postoperative glaucoma medications, graft detachment rate, and graft survival rate. All the recorded outcomes were evaluated at 3 and 12 months.

The postoperative interventions recorded at any time points were as follows: any unscheduled increase in topical steroid medication (transplant rejection); air injection into the AC (transplant detachment); additional required surgery (graft dislocation); additional glaucoma medications (intraocular pressure control); and repeat corneal transplantation (graft failure).

### 2.4. Data Analysis

Data analysis was performed using SPSS (IBM SPSS Statistics v. 26.0.0.0). Normality was checked using the Kolmogorov-Smirnov test. Descriptive statistics were expressed as a raw number and percentage and as the mean ± standard deviation for categorical and continuous variables, respectively.

The differences between the group means were evaluated using the t-test and one-way analysis of variance (one-way ANOVA) for parametric, unmatched, continuous variables. In particular, Welch’s t-test was applied due to the significant difference in sample size between the groups. The Wilcoxon matched-pairs signed rank test was used to compare non-parametric, matched, continuous variables. The Kruskal-Wallis test was used to compare non-parametric, unmatched, continuous variables. The *χ*^2^ test or Fisher exact test for proportions were used, as appropriate, for categorical variables. Multiple comparisons were adjusted using the Bonferroni-Holm method and Dunnett’s Test.

Graft survival analysis was conducted using the Kaplan-Meier estimator. The log-rank test was used to evaluate any differences in survival rates among the groups. A Cox proportional hazards model was applied to investigate the association between survival time and possible predicting variables.

All tests were 2-sided and a *p* value of less than 0.05 was regarded as proof of statistical significance.

## 3. Results

### 3.1. Patient Demographics

A total of 80 DSAEKs and 16 DMEKs procedures were recorded in 78 eyes (63 DSAEK and 15 DMEK) of 73 patients (59 DSAEK and 14 DMEK). Based on the different sample size, a non-parametric test and post-hoc analysis were performed for each comparison to limit the numerosity bias effect. None of the patients were lost to follow-up. The preoperative demographic data are summarised in Table 1.

In both groups, the primary surgical indication was bullous keratopathy: 87.5% and 76.3% in the DMEK and the DSAEK groups, respectively. Fuchs endothelial dystrophy (FED) affected only one eye in both groups (6.3% DMEK; 1.3% DSAEK). Previous graft failure (DMEK or DSAEK) was the indication in 1 eye in the DMEK group (6.3%) and in 18 eyes in the DSAEK group (22.5%).

### 3.2. Visual Outcomes

BCVA improved after surgery in both the DMEK and DSAEK groups (Table 2). In the DMEK group, BCVA increased from 1.31 ± 1.11 LogMAR preoperatively to 0.64 ± 0.95 LogMAR at the 3-month follow-up, while in the DSAEK group, BCVA improved from 1.60 ± 1.15 LogMAR preoperatively to 1.06 ± 1.07 LogMAR at 3 months. At 12 months, BCVA remained better than the baseline in both groups, with 0.79 ± 1.04 LogMAR in the DMEK group and 1.16 ± 1.12 LogMAR in the DSAEK group. However, there was no statistically significant difference in BCVA between DMEK and DSAEK at any follow-up time point (*p* > 0.05), indicating comparable visual outcomes between the two techniques.

### 3.3. Intraocular Pressure

The mean preoperative IOP values were 11.6 ± 4.7 mmHg and 13.4 ± 3.9 mmHg for the DMEK and DSAEK groups, respectively (*p* = 0.3196). In both groups, no statistically significant differences were found between the preoperative mean IOP values compared to both 3 and 12 months postoperatively (DMEK: *p* = 0.0561 DSAEK: *p* = 0.0828). In both cohorts, nearly 60% of the patients were using topical anti-glaucoma medications preoperatively (Table 1). To control the postoperative IOP, 10.5% of the patients in the DMEK group and 13.3% of the patients in the DSAEK group required additional anti-glaucoma agents (*p* = 0.6460). Postoperatively, we found significantly lower IOP values in the DMEK group compared to the DSAEK group at 3 months and 12 months (Table 2). No pupillary block episodes were observed in either group at any timepoints.

### 3.4. Graft Detachment, Failure, and Survival

Visually significant graft detachment requiring further intervention was observed in 5 out of 16 (31.3%) DMEK cases and in 18 out of 80 (22.5%) cases in the DSAEK group (*p* = 0.4541). These figures are in line with currently available data [16,17]. (Table 3). At our institution, DMEK graft rebubbling is usually performed in cases of partial graft detachment involving more than 1/3 of the graft surface or graft detachment involving the visual axis causing visual deterioration. All the DMEK rebubbling procedures were successfully performed at the slit lamp [11]. No DMEK cases required secondary intervention after the primary graft rebubbling. A minor DMEK detachment (less than 1/3) was observed, and it reattached spontaneously with time (1 out of 16, 6.3%).

In the DSAEK group, 2 out of 18 cases required observation due to a small graft detachment. Six large graft detachments with no dislocation underwent rebubbling performed in the operating theatre and 10 eyes needed primary graft repositioning for graft dislocation. Of the 6 re-bubbled cases, 2 were unsuccessful and required a second rebubbling procedure.

Allograft rejection was observed in only 1 DMEK case (6.3%), within 1 year after surgery. Despite an intensive regimen of topical steroids, the graft subsequently failed. In the DSAEK group, the allograft rejection rate was noted to be as high as 18.8% (15 of 80 eyes) within the first 12 months (*p* = 0.2207).

Within the first year of corneal transplant, graft failure was observed in 2 out of 16 eyes (12.5%) that had undergone DMEK surgery and 24 out of 80 eyes (30.0%) in the DSAEK group (*p* = 0.1504).

**Figure 1:**
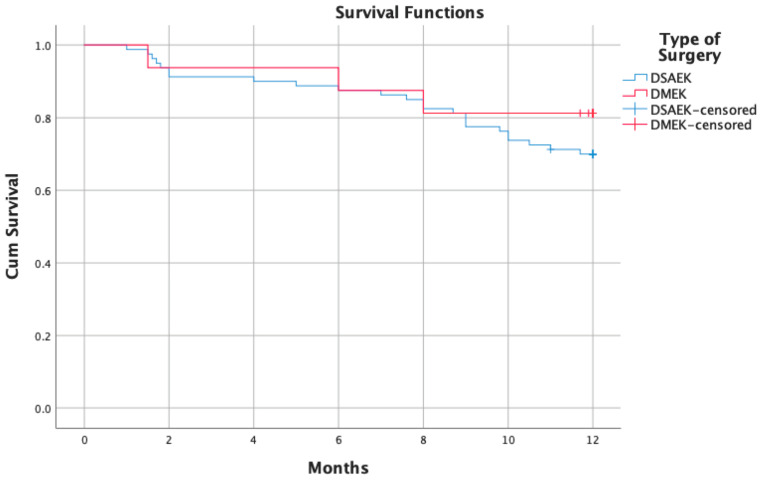
Kaplan-Meier survival curve demonstrating the 12-month cumulative survival rate of Descemet membrane endothelial keratoplasty grafts compared with Descemet stripping automated endothelial keratoplasty grafts in eyes with prior glaucoma surgery. (DMEK: Descemet membrane endothelial keratoplasty; DSAEK: Descemet stripping automated endothelial keratoplasty).

Kaplan-Meier curves demonstrated a cumulative graft survival rate at 1 year of 71.9%. Stratifying the selected cohort according to the type of surgery (DMEK vs. DSAEK), a survival rate of 81.3% in the DMEK group and of 70% in the DSAEK one was found (*p* = 0.431) (Figure 1). The Cox regression model failed to identify any possible factor able to alter graft failure survival rates.

Subsequently, redo keratoplasty was performed for the 2 failed DMEKs: 1 patient underwent DSAEK surgery and 1 underwent penetrating keratoplasty. Nine out of 24 failed DSAEK grafts (37.5%) had repeated keratoplasty within the first year (3 redo-DSAEK, 4 penetrating keratoplasty, 2 Boston-K-Pro procedures).

One DSAEK patient developed retinal detachment 1 month following surgery and required pars plana vitrectomy with a silicone oil implant.

## 4. Discussion

This study compared the clinical outcomes of DMEK and DSAEK surgery in eyes which had previously undergone glaucoma filtering surgery (trabeculectomy, tube shunt, or both). Overall, we did not find any difference at the baseline between eyes undergoing DMEK or DSAEK in any of the analysed variables, including previous glaucoma surgery. While the percentage of eyes with a history of trabeculectomy was higher in the DSAEK group, the percentage of eyes with a history of both trabeculectomy and GDD was higher in the DMEK group. Hence, a homogeneous distribution of the two types of surgery may be assumed between the two groups.

In such cases, DMEK surgery is considered a technically challenging procedure. Two main intraoperative difficulties have been seen to hamper the correct and efficient unfolding of the graft. First of all, the presence of iris synechiae or of an incomplete iris plane or tube substantially alters the AC anatomy. Additionally, the presence of a drainage pathway can complicate the retention of the air or gas bubble within the anterior chamber.

Thus, DSAEK may be considered a safer approach, as graft unfolding is believed to be easier with a thicker donor material [17,18,19]. Our findings, however, showed a relatively high rate of rebubbling in the DMEK group, with 31.3% of cases requiring this intervention. While this percentage is higher when compared to DSAEK (22.5%), it is important to note that rebubbling following DMEK is often performed at the slit lamp, making it a less invasive and more straightforward procedure. Furthermore, DSAEK cases were also complicated by graft detachment, which required additional interventions to ensure proper graft attachment. Finally, despite the greater technical complexity of DMEK and the increased rate of rebubbling, these intraoperative difficulties did not result in worse outcomes when compared to DSAEK. Although DMEK presents more challenges due to the thinner graft, the procedure still demonstrated comparable long-term results, suggesting that the technical difficulties did not adversely affect the overall surgical outcomes.

The technical ease of DSAEK is not accompanied by equally appreciable visual outcomes than the ones observed in DMEK. In fact, the analysis of data coming from similar cohorts confirms better visual outcomes in DMEK- than DSAEK-operated eyes [16]. While BCVA improvement at both 3 and 12 months did not appear to be statistically significant, it is worth noting that the mean values in the DMEK group were strikingly lower than those observed in DSAEK eyes. In fact, as is widely discussed in the literature, DMEK-operated eyes have not only a better postoperative BCVA, but also a faster visual recovery than DSAEK, probably due to the thinner transplanted graft [20].

The difference in IOP between DMEK and DSAEK in this cohort was statistically significant both at 3 and 12 months postoperatively (*p* = 0.0272 and 0.0447, respectively), coherently to the previously described higher prevalence of raised IOP and glaucoma after DSAEK compared with DMEK [2,21]. However, in a recent long-term retrospective comparative case series, including patients with previous trabeculectomy or glaucoma drainage device implantation, who underwent either DMEK or DSAEK, no differences in postoperative IOP elevation were observed between the groups [16]. This discrepancy might be explained by the unequal numerosity of the DMEK vs. DSAEK groups in our cohort. Another possible explanation for this difference lies in the central corneal thickness (CCT), which is typically higher in DSAEK than in DMEK. An elevated CCT can artificially increase IOP readings due to the thicker corneal tissue in DSAEK, making IOP measurements less accurate and potentially overstating the actual IOP in these patients. This factor may contribute to the observed differences in IOP between the two groups, considering that both groups showed a similar increase in the number of lowering IOP medications.

It must be considered that the postoperative IOP increase was effectively managed with the prompt use of additional glaucoma medications. As a result, no statistically significant increase was observed in the postoperative IOP compared to the preoperative one. Specifically, up to 10% of the patients in the DMEK group and 13.3% of the patients in the DSAEK one required additional IOP lowering drops (*p* = 0.6460). Similar results were obtained by Quek et al. [22] and by Phillips et al. [23], who analysed DSAEK-related outcomes in eyes previously treated with glaucoma filtering surgery. Further studies should explore alternative methods of IOP control which promise prolonged efficacy and reduced local side effects in patients with concurrent corneal conditions, potentially improving IOP control in the EK population [24].

Overall, the incidence of graft detachment has been reported to be higher in DMEK-compared to DSAEK-operated eyes [5,25]. In our cohort, we found that the graft detachment rate was not significantly different between the two groups. However, the incidence of DMEK total graft detachment and associated dislocation appeared to be lower than the one observed after DSAEK. All the detachments were successfully treated with slit-lamp rebubbling in the DMEK group, with no cases of graft dislocation. Conversely, all the DSAEK rebubbling procedures (for partial detachment) were performed in the operating theatre, with 33% of the eyes (2 out of 6 graft detachments) requiring a further graft repositioning for associated graft dislocation after the first rebubbling. Notably, the management of a detached and dislocated DSAEK graft poses a great burden on patients and clinical resources as repositioning must be performed in an operating theatre. The rebubbling rate observed in our study appeared to be slightly higher than the one observed in the studies of Lin et al. and in Birbal et al. [19,26]. This difference may be due to the presence of more challenging cases in our cohort (multiple glaucoma surgeries).

In our series, the prevalence of graft rejections and graft failures at one year was lower in the DMEK group than in the DSAEK one (Kaplan-Meier Survival curve: DMEK: 81.3%; DSAEK: 70%), in line with data already present in the literature (Table 3). In a recently published paper, Alshaker et al. proposed a 1-year survival probability for DMEK and DSAEK grafts of 75% [16]. Similarly, Sorkin et al., evaluating the 4-year outcomes of DMEK in eyes with previous glaucoma surgery, reported a cumulative 1-year survival rate of 75% [18]. Conversely, Birbal et al. [19] reported a higher graft survival rate in 23 DMEK eyes (89%) which had previously undergone glaucoma drainage surgery [19].

Compared to DSAEK, DMEK requires advanced surgical skills and has a steeper learning curve. We believe surgical expertise or newer “endothelium-in” graft insertion techniques can help spearhead DMEK in such complex cases [27,28,29]. These newer endothelium-in techniques allow the graft to be inserted into the eye in the correct orientation with the endothelium facing downwards. Following insertion, the graft starts to unfold with minimal manipulation, thus reducing the risk of an upside-down graft and minimising further graft manipulation and endothelial cells loss.

It is important to consider the limitations of our study when interpreting the results. One key limitation is the different sample sizes of the two groups analysed, which may have reduced the statistical power and external validity. However, we attempted to address this by using non-parametric tests and post-hoc analysis for multiple comparisons. Additionally, selection bias may have played a role, as more complex cases may have been treated with DSAEK instead of DMEK due to the perceived ease and familiarity of the former among corneal surgeons. While DMEK is increasingly being performed even in more complex cases, as surgeons gain experience and confidence with the technique [30], the historical preference for DSAEK in challenging scenarios could still introduce bias into the analysis, particularly in cases where DMEK may not have been considered an option in earlier years.

Another limitation is the retrospective nature of the study. However, retrospective studies may offer a more accurate representation of real-world scenarios. In fact, clinical trials and prospective studies have been acknowledged for their own pitfalls, such as narrow inclusion and exclusion criteria, strict treatment and follow-up protocols, and a lack of representation of diverse ethnic backgrounds, which can limit their ability to describe the complexity and diversity of clinical populations. Additionally, our study’s sample size, particularly regarding the different types of glaucoma (e.g., open-angle, angle-closure), was not sufficient to allow for a meaningful subgroup analysis. This limitation restricted our ability to explore how these different glaucoma types might impact surgical outcomes or complication rates between DMEK and DSAEK. Despite these limitations, the concordance of our findings with those previously reported in the literature lends support to the robustness of our analysis.

Our data confirm that, although more challenging, DMEK is a feasible technique for restoring endothelial function in complicated eyes, such as in cases of previous filtering glaucoma surgery (trabeculectomy and/or glaucoma drainage device). Though graft survival rates appear to be lower than the ones observed in healthy eyes, DMEK provides satisfactory visual outcomes, with a lower risk of postoperative IOP increase, rebubbling, and graft rejection than the ones registered in the case of DSAEK.

Further prospective randomised controlled studies will be required to better elucidate the true effect on long-term visual outcome.

## Figures and Tables

**Table 1:** Patients’ features.

Features	DMEK	DSAEK	*p* Values
Patient			
Number of procedures/eyes/patients	16/15/14	80/63/59	
Recipient age, years (mean ± SD)	65.7 ± 16.4	62.0 ± 16.2	0.4560
Sex			
Female, *n* (%)	7 (43.8)	34 (42.5)	0.8620
Male, *n* (%)	9 (56.3)	46 (57.5)	
Pre-op BCVA mean ± SD	1.31 ± 1.11	1.60 ± 1.15	0.3196
Lens status			
PC-IOL, *n* (%)	15 (93.8)	68 (85.0)	0.3504
AC-IOL, *n* (%)	1 (6.3)	12 (15.0)	
Indication for surgery			
Bullous keratopathy, *n* (%)	14 (87.5)	61 (76.3)	
Fuchs endothelial dystrophy, *n* (%)	1 (6.3)	1 (1.3)	0.1578
Failed previous transplant, *n* (%)	1 (6.3)	18 (22.5)	
Type of glaucoma			
Primary open-angle glaucoma, *n* (%)	6 (37.5)	29 (36.3)	0.8160
Secondary glaucoma, *n* (%)	6 (37.5)	18 (22.5)	
Angle-closure glaucoma, *n* (%)	1 (6.3)	4 (5.0)	
Congenital glaucoma, *n* (%)	1 (6.3)	8 (10.0)	
Surgeon grade			
Consultant, *n* (%)	8 (50.0)	44 (55.0)	0.7870
Fellow, *n* (%)	8 (50.0)	36 (45.0)	
Trabeculectomy, *n* (%)	1 (6.3)	12 (15.0)	0.5150
Tube(s)			
0, *n* (%)	11 (68.8)	42 (52.5)	
1, *n* (%)	4 (25.0)	36 (45.0)	0.2840
2, *n* (%)	1 (6.3)	2 (2.5)	
Trabeculectomy and tube(s), *n* (%)	9 (56.3)	30 (37.5)	0.1633
Previous PK, *n* (%)	2 (12.5)	18 (22.5)	0.3690
Glaucoma drops prior to EK			
No	6 (37.5)	32 (40.0)	0.8980
Yes (agents)			
1	3 (18.8)	20 (25.0)	
2	5 (31.3)	16 (20.0)	
3	2 (12.5)	9 (11.3)	
4	0 (0)	1 (1.3)	
5	0 (0)	2 (2.5)	

DMEK = Descemet membrane endothelial keratoplasty; DSAEK = Descemet stripping automated endothelial keratoplasty; Pre-Op = preoperative; PC-IOL = posterior chamber intraocular lens; AC-IOL = anterior chamber intraocular lens; PK = penetrating keratoplasty; EK: endothelial keratoplasty.

**Table 2:** Preoperative and postoperative best-corrected visual acuity, intraocular pressure, and glaucoma treatment.

	DMEK	DSAEK	*p* Values
LogMAR BCVA, mean ± SD			
Preoperative	1.31 ± 1.11	1.60 ± 1.15	0.3196
3m FU	0.64 ± 0.95	1.06 ± 1.07	0.1199
12m FU	0.79 ± 1.04 ^1^	1.16 ± 1.12 ^2^	0.2844
IOP in mmHg, mean ± SD			
Preoperative	11.6 ± 4.7	13.4 ± 3.9	0.1638
3m FU	10.3 ± 3.3	14.1 ± 6.9	0.0272
12m FU	10.3 ± 2.8 ^3^	15.3 ± 4.7 ^4^	0.0447
Additional glaucoma drops postoperatively, *n* (%)	2 (10.5)	19 (13.3)	0.6460

DMEK = Descemet membrane endothelial keratoplasty; DSAEK = Descemet stripping automated endothelial keratoplasty; BCVA = best spectacle visual acuity; SD= standard deviation; IOP = intraocular pressure; FU = follow-up; m = month. ^1^ ANOVA-DMEK-BCVA *p* < 0.0001; ^2^ ANOVA-DSAEK-BCVA *p* < 0.0001; ^3^ ANOVA-DMEK-IOP *p* = 0.0561; ^4^ ANOVA-DSAEK-IOP *p* = 0.0828.

**Table 3:** Postoperative complications.

Complications	DMEK 16 *n* (%)	DSAEK 80 *n* (%)	*p* Values
Pupillary block	0 (0)	0 (0)	
Hypotony	0 (0)	2 (2.5)	
Graft detachment	5 (31.3)	18 (22.5)	0.4541
Rebubbling	5 (31.3)	6 (7.5) *	0.0173
Repositioning	0 (0)	10 (12.5)	0.1351
Allograft rejection ^§^	1 (6.3)	15 (18.8)	0.2207
Graft failure ^§^	2 (12.5)	24 (30.0)	0.1504
Re-keratoplasty ^§^	2 (12.5)	9 (11.25)	0.8331
DMEK	0 (0)	0 (0.0)	
DSAEK	1 (6.3)	3 (3.75)	
PK	1 (6.3)	4 (5.0)	
Boston KPro	0 (0)	2 (2.5)	
RRD	0 (0)	1 (1.25)	

DMEK = Descemet membrane endothelial keratoplasty; DSAEK = Descemet stripping automated endothelial keratoplasty; PK = Penetrating keratoplasty; Boston KPro = Boston Keratoprosthesis. RRD = Rhegmatogenous retinal detachment. ^§^ In the first 12 months. * 3 DSAEK rebubbling failed and required subsequent repositioning.

## Data Availability

Available upon request to corresponding authors.
